# Using Multiscale Spatial Models to Assess Potential Surrogate Habitat for an Imperiled Reptile

**DOI:** 10.1371/journal.pone.0123307

**Published:** 2015-04-27

**Authors:** Jennifer M. Fill, Jayme L. Waldron, Shane M. Welch, J. Whitfield Gibbons, Stephen H. Bennett, Timothy A. Mousseau

**Affiliations:** 1 Department of Biological Sciences, University of South Carolina Columbia, Columbia, South Carolina, United States of America; 2 Department of Biological Sciences, Marshall University, Huntington, West Virginia, United States of America; 3 Savannah River Ecology Laboratory, Aiken, South Carolina, United States of America; 4 South Carolina Department of Natural Resources, Columbia, South Carolina, United States of America; Università degli Studi di Napoli Federico II, ITALY

## Abstract

In evaluating conservation and management options for species, practitioners might consider surrogate habitats at multiple scales when estimating available habitat or modeling species’ potential distributions based on suitable habitats, especially when native environments are rare. Species’ dependence on surrogates likely increases as optimal habitat is degraded and lost due to anthropogenic landscape change, and thus surrogate habitats may be vital for an imperiled species’ survival in highly modified landscapes. We used spatial habitat models to examine a potential surrogate habitat for an imperiled ambush predator (eastern diamondback rattlesnake, *Crotalus adamanteus*; EDB) at two scales. The EDB is an apex predator indigenous to imperiled longleaf pine ecosystems (*Pinus palustris*) of the southeastern United States. Loss of native open-canopy pine savannas and woodlands has been suggested as the principal cause of the species’ extensive decline. We examined EDB habitat selection in the Coastal Plain tidewater region to evaluate the role of marsh as a potential surrogate habitat and to further quantify the species’ habitat requirements at two scales: home range (HR) and within the home range (WHR). We studied EDBs using radiotelemetry and employed an information-theoretic approach and logistic regression to model habitat selection as use vs. availability. We failed to detect a positive association with marsh as a surrogate habitat at the HR scale; rather, EDBs exhibited significantly negative associations with all landscape patches except pine savanna. Within home range selection was characterized by a negative association with forest and a positive association with ground cover, which suggests that EDBs may use surrogate habitats of similar structure, including marsh, within their home ranges. While our HR analysis did not support tidal marsh as a surrogate habitat, marsh may still provide resources for EDBs at smaller scales.

## Introduction

Wildlife conservation and management strategies rely strongly on knowledge of species-habitat relationships. Species exploit resources from habitat patches that vary as ecological communities interact with human land use, geomorphology, and geo-physical processes (e.g., agricultural fields, tidal marshes). Different patch types may be used to support various characteristics of a species’ niche [1]. Alternatively, an animal may exploit substitutable resources in multiple patches [2]. Thus, habitat patches that meet similar niche requirements, such as trophic interactions and vegetation structure, may allow a species to use additional landscape patches as surrogate habitats. This is a particularly relevant consideration for conservation scenarios that may be constrained by an animal’s limited geographic distribution, specialized niche requirements, the loss or alteration of native habitats, or the need to meet multiple management objectives. In evaluating conservation and management options for a species, practitioners might consider surrogate habitats when estimating available habitat or modeling a species’ potential distribution based on suitable habitats, especially when indigenous environments are rare or degraded [3–5]. The ability of a landscape patch to serve as surrogate habitat may enhance or broaden conservation and management options.

Animal home range behavior (establishment of and space use within a restricted area) is an informative spatial concept underlying plans or protocols that target habitat conservation for a species. By linking an animal’s repeated use of an area with the resources it needs for survival and reproduction (“habitat”) [6–8], home range behavior provides a basis for modeling suitable habitat and investigating the potential for habitats to function as surrogates. However, an animal’s establishment of a home range by developing a cognitive map of resources [8], and movement within that home range, are influenced by habitat variables that may be scale-dependent [9–12]. Thus, examining an animal’s home range habitat selection at both a landscape scale and within the home range (second- and third-order selection, [13]) provides a more accurate basis for habitat evaluation at multiple scales [10, 11, 13], consequently improving the accuracy of models involving optimal and surrogate habitats.

A thorough evaluation of habitat conservation options is especially important in threatened ecosystems, where native habitats are fragmented or lost due to anthropogenic change. Species in these systems are likely to increasingly depend on surrogate habitats. In the southeastern United States, loss of longleaf pine (*Pinus palustris*) ecosystems is an immediateconcern [14–17]. Historically, these fire-maintained savannas and woodlands dominated approximately 37 million hectares across the southeastern Coastal Plain [14]. Following European settlement, they experienced pronounced alterations and declines due to human land-use practices and fire exclusion [15], and now occur over less than five percent of their former range [16, 17]. Among the most imperiled ecosystems in North America [14, 16], they exhibit notably high rates of floral and faunal endemism [18, 19]. A number of threatened or endangered vertebrate species depend on longleaf habitats, including the flatwoods salamander (*Ambystoma cingulatum*), gopher tortoise (*Gopherus polyphemus*), and red-cockaded woodpecker (*Picoides borealis*).

The eastern diamondback rattlesnake (*Crotalus adamanteus*; EDB) is a cryptic predator indigenous to longleaf savanna and woodland habitats [18, 20–22]. Its historical range extended from North Carolina to southern Florida, and west to Missisippi and southeastern Louisiana [23]. Loss of pine savanna habitats has been suggested as the principal cause of the species’ widespread decline [23], and it is listed as a species of concern in all but two states throughout its range, as state-endangered in North Carolina, and has been extirpated from Louisiana [23]. Consequently, few viable extant EDB populations remain throughout its former range and the species is in review for listing under the Endangered Species Act [24]. Because the secretive nature of many reptiles makes it difficult to monitor population status, information about their natural history, including habitat use, is essential in developing conservation and management strategies [25].

Although pine savannas have declined, EDBs in coastal tidewater areas may be using marsh as surrogate habitat. The EDB is principally associated with open canopy, pine savanna habitats at local and regional scales [21–23, 26, 27]. These are open-canopy ecosystems with an overstory dominated by pines and a dense herbaceous groundcover. Pine savannas burn frequently, provide shelters for EDBs (gopher tortoise and armadillo (*Dasypus novemcinctus*) burrows, fallen trees, burned-out stumps), and support large ground-dwelling mammalian prey items such as cotton rats (*Sigmodon hispidus*) and fox squirrels (*Sciurus niger*) [23]. Although EDBs are less commonly associated with extensive low-lying or frequently flooded areas [23], it has been suggested that marshes are important surrogate habitat in coastal regions [28]. Marshes have a vegetation structure similar to pine savannas (open canopy, low groundcover) and support suitable prey items as a substitutable resource [28]. Given the extensive decline of pine savannas [15–17], it is important to assess the potential role of marsh as suitable habitat for EDBs when resource managers in tidewater regions are attempting to model available habitat for EDBs at broad scales. This study represents the first attempt to examine habitat selection where marsh is an available component of the landscape.

The purpose of this study was to examine EDB habitat selection in the Coastal Plain tidewater region with respect to vegetation and topography. We determined habitat selection by comparing EDB habitat use relative to availability [13]. Our objectives were to 1) evaluate the role of marsh as a potential surrogate habitat and 2) further quantify the species habitat requirements at two scales: home range (HR) and within the home range (WHR). We hypothesized that EDBs in coastal areas select pine savanna and marsh habitats; we therefore predicted that EDBs would exhibit disproportionate use of pine savanna and marsh relative to their availability. We further predicted that EDBs would display a positive association with groundcover and a negative association with canopy cover. Additionally, we hypothesized that these habitat associations would be apparent at both scales of selection, but that selection for topographic characteristics would only be apparent at the HR scale.

## Materials and Methods

### Study Area

We conducted this study on privately-owned property in Colleton County in the southeastern South Carolina coastal plain. The study area was delineated in ArcGIS 10.1 [29] as the property boundary, encompassing c. 4,600 ha of mixed upland longleaf, loblolly (*Pinus taeda*), and slash pine (*Pinus elliottii*), mixed pine-hardwood, and hardwood bottom habitats. Fields and wildlife food plots were interspersed throughout the property. The uplands were almost entirely bordered by tidal marshes and 19^th^ century rice impoundments now managed for waterfowl. For two decades and ongoing, the property was part of the ACE Basin (the area at the confluence of the Ashepoo, Combahee, and Edisto Rivers) Conservation Project, and was managed primarily for bobwhite quail (*Colinus virginianus*) with high-frequency prescribed fire and timber harvests to maintain and restore open-canopy pine savannas.

### Radiotelemetry

Rattlesnakes (non-gravid females, *n* = 3; males, *n* = 5) were captured and surgically implanted with radio transmitters (SI-2, 11–13 g, Holohil Systems, Carp, Ontario) as described by Waldron et al. (2008). We used radiotelemetry to locate EDBs during four field seasons: May 2006–April 2008 and July 2011–January 2013. We radiotelemetrically monitored each of seven animals for one year during the two tracking periods, and one male was tracked for 14 months. Each animal was located at least once per week during the active season (March–October) and biweekly in the inactive season (November–February) using a Telonics TR-4 radio receiver and an RA-14k ‘H’ antenna (Telonics, Inc., Mesa, AZ). Using homing techniques, each snake location was visually identified in the field (eliminating triangulation error), and recorded using a handheld GPS (Magellan, Explorist 500; or Trimble Pro XR, Sunnyvale, CA, USA).

### Ethics Statement

Research was conducted in strict accordance with the recommendations in the Guide for the Care and Use of Laboratory Animals of the National Institute of Health. We obtained protocol approvals (1724-100370-061011) and (2011-100248-052311) from the University of South Carolina Institutional Animal Care and Use Committee. Isofluorane was used to anesthetize snakes during surgery in accordance with approved procedures. We also obtained University of Georgia permits (A2006-10175 and A2009 6–119).

### Home Range Delineation

We used the Geospatial Modelling Environment (GME) in ArcGIS to generate 95% kernel density estimates of home ranges for each individual [30]. We used the plug-in bandwidth estimator as a conservative estimate of home range area, decreasing the likelihood of overestimation [31, 32]. Although the least-squares cross-validation estimator has been recommended as the most accurate smoothing parameter, it is not well-suited for datasets in which animals use the same location multiple times [30] Autocorrelation can lead to inaccurate home range estimates, but increasing the number of locations can decrease this bias [33]. Kernel density estimators are much less sensitive to autocorrelation than other home range estimation methods [33, 34]. They have also been shown to be robust to variable sampling intensities, as long as the sampling duration is consistent among individuals [35, 36]. Inconsistency in kernel density estimates are most often associated with home range size; however, in this study we were concerned with spatial ecology within the home range, making kernel density estimators appropriate [37]. Each snake was tracked for one year; for the male tracked for 14 months, we used only locations within one year from the start of telemetry for each individual to ensure consistent sampling duration. Each snake had 29–55 fixes for use in the analysis (*n* = 281).

### Generation of Use and Random Locations

For snakes sampled once per week, we considered each weekly location within the one-year sampling duration as an indication of habitat selection by that animal. Eastern diamondback rattlesnakes are ambush predators, frequently exhibiting limited movement for days at a time [38]. When successive locations were within less than 15 m of each other and temporally separated by less than one week, we randomly chose one of those locations as an independent observation of “use.” We used randomly generated points to assess habitat availability for both scales of analysis. Because the number of random locations should be large relative to the number of used locations when examining selection [39], w e used the random point generator in ArcGIS 10.1 to generate random locations within the home range in a 2:1 ratio for each snake (total random *n* = 562). For each snake, we generated twice as many HR random locations across the landscape as WHR locations. Random HR locations (total random *n* = 1148) were restricted to within the boundary of the study area, which included extensive representation of all cover types, but not the river channels. To minimize plot overlap (see next section) and maintain compatibility with land cover spatial resolution, all random locations were separated by a minimum distance of 30 m.

### Vegetation and Topographic Metrics

We characterized vegetation and topographic variables within a 15-m radius plot of each snake and random location in ArcGIS (S1 Table). We chose a 15-m radius to account for imprecision in the locations that were obtained using the handheld GPS (7-m accuracy) [40].

We obtained small footprint multiple return LiDAR (Light Detection and Ranging) from the NOAA Coastal Services Center South Carolina LiDAR Mapping. This dataset included raw LAS files for Colleton County that were collected using a Leica ALS-50 sensor between 2 February 2007 and 23 March 2007. We interpolated ground returns using second power Inverse Distance Weighted interpolation in the 3D Analyst GIS environment to obtain a Digital Elevation Model (DEM) with 1-m resolution [41, 42]. The DEM heights were then assigned to all point cloud data, allowing the computation of height above ground for every data point [42]. We extracted LiDAR measurements of canopy cover and ground cover to describe structural information for each snake and random plot. Canopy cover (CANCOV) was measured by redefining closed canopy returns as only the ones equal to or greater than two meters and dividing the total number of these returns in each plot by all discrete returns in the same plot, including ground returns [42], [43]. Ground cover (GROUNDCOV) was measured as the proportion of returns between 0.5 and 2 m in height [42].

We used 2006 National Land Cover Classification categories at 30-m resolution to define the dominant vegetation cover type in each plot (COVER) [44]. We compared NLCD categories to 2008 high-resolution aerial photographs and knowledge of the study area. We combined NLCD cover types to define relevant cover categories for this study (Table 1). Both woody wetlands (WW) and herbaceous wetlands (HW) categories encompassed marshy areas and brackish impoundments. Woody wetlands were primarily mesic lowlands in the study area interior but included landscape patches adjacent to marsh. Herbaceous wetlands primarily characterized marsh and impoundments. Since the NLCD developed open space category was primarily dirt or gravel roads through pine savanna habitats, we included this in pine savanna (PS). We used GME to identify the cover type of the greatest proportion in each plot.

**Table 1:** Cover types (derived from 2006 National Land Cover Classification categories) for eastern diamondback habitat selection analyses in the South Carolina Coastal Plain tidewater region.

Cover type	NLCD 2006 categories
Forested (FOR)	Deciduous, evergreen, and mixed forest
Wildlife food plot (FP)	Grassland/herbaceous, pasture/hay, cultivated crops
Herbaceous wetlands (HW)	Emergent herbaceous wetlands
Open water (OW)	Open water
Pine savanna (PS)	Shrub/scrub, developed open space
Woody wetlands (WW)	Woody wetlands

Finally, we used Jenness Tools [45] to derive a raster of aspect in degrees for the study area (ASP). We used GME to extract aspect and elevation (ELEV; from the DEM) for each plot. We linearized aspect using the equation: [1-cosine(aspect in radians)] + [1-sine(aspect in radians)] so that northeasterly aspects had low values and southwesterly aspects had high values [46, 47]. We standardized elevation so values were bound between zero and one.

### Statistical Analysis

We used binomial logistic regression in PROC GLIMMIX [48] to compare used versus random locations (use vs. availability) at the HR and WHR scales. We first developed a set of a priori candidate models (Tables 2 and 3) [49]. We included LiDAR-derived canopy cover and groundcover, and cover type as vegetative predictors. Aspect and elevation served as topographic predictors. We did not have enough females to justify inclusion of sex as a predictor. We examined correlation coefficients among predictor variables to check for collinearity; those with r > 0.7 would be excluded. No predictors were correlated. We ran each model as logistic regression with the Laplace approximation [50], including snake as a random factor. Since our hypotheses addressed canopy cover and cover independently, we used the global model only to examine fit using Pearson’s χ^2^/df. We retained candidate models with an Akaike’s Information Criterion difference (ΔAIC) of < 2.00 for statistical inference. We used 95% confidence intervals to determine the sign of parameter estimates; those that included zero were indeterminate.

**Table 2:** Candidate models for eastern diamondback rattlesnake habitat selection at the home range scale (*n* = 1429) in the South Carolina Coastal Plain tidewater region.

Rank	Model	*k*	AIC	ΔAIC	*w*_*i*_
1	**COVER+ASP+ELEV**	8	1300.44	0	1.00
2	CANCOV+GROUNDCOV+ASP+ELEV	5	1335.67	35.23	0.00
3	COVER	6	1351.38	50.94	0.00
4	CANCOV+ASP	3	1354.27	53.83	0.00
5	CANCOV+GROUNDCOV+ASP	4	1354.46	54.02	0.00
6	CANCOV	2	1396.89	96.45	0.00
7	CANCOV+GROUNDCOV	3	1397.27	96.83	0.00

Included are the number of estimated parameters (*k*), Akaike’s information criterion (AIC), Akaike’s information criterion differences (ΔAIC), and Akaike weights (*w*_*i*_). Models retained for statistical inference are in bold.

**Table 3:** Candidate models for eastern diamondback rattlesnake habitat selection at the within-home range scale in the South Carolina Coastal Plain tidewater region (*n* = 917).

Rank	Model	*k*	AIC	ΔAIC	*w*_*i*_
1	**COVER+ASP+ELEV**	8	1098.12	0	0.45
2	**CANCOV+GROUNDCOV+ASP**	4	1098.62	0.50	0.35
3	CANCOV+GROUNDCOV+ASP+ELEV	5	1100.53	2.41	0.13
4	CANCOV+ASP	3	1101.80	3.68	0.07
5	COVER	6	1124.22	26.10	0.00
6	CANCOV+GROUNDCOV	3	1130.17	32.05	0.00
7	CANCOV	2	1132.68	34.56	0.00

Included are the number of estimated parameters (*k*), Akaike’s information criterion (AIC), Akaike’s information criterion differences (ΔAIC), and Akaike weights (*w*_*i*_). Models retained for statistical inference are in bold.

## Results

### Home Range Selection

**Fig 1:**
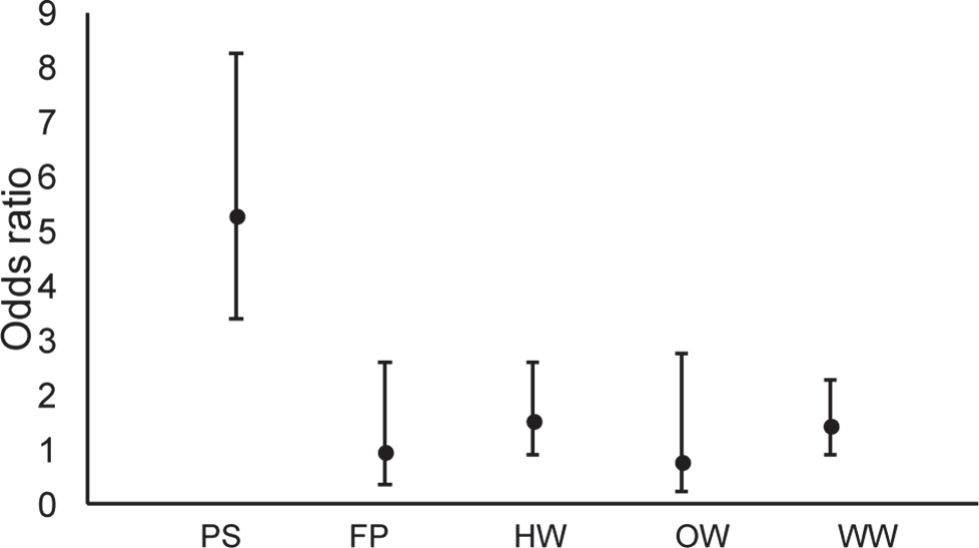
Odds ratios by cover type for eastern diamondback rattlesnake habitat selection at the home range scale in the South Carolina Coastal Plain tidewater region. Reference = forest; PS = pine savanna, FP = wildlife food plot, HW = herbaceous wetland, OW = open water, WW = woody wetland.

Fit statistics indicated good model fit in both scales (Pearson χ^2^/df: HR = 1.05; WHR = 1.00). At the HR scale, only one candidate model received support. Cover type, aspect, and elevation were important predictors of EDB habitat selection (Table 2). The next highest-ranking candidate model had a ΔAIC much greater than 2.00, indicating little empirical support. Both vegetation and topography had significant influence on HR selection (Table 4). EDBs exhibited significantly negative associations with all land cover classes other than PS (Fig 1), and selected locations that were oriented in a southwesterly direction (Table 4). All three predictors were equally important, with each parameter contributing 100% to model weights. Odds ratios indicated that the odds of rattlesnakes using pine savanna were 5.0, 5.5, 3.5, 6.9, and 3.7 times higher than those of rattlesnakes using forest, food plot, herbaceous wetland, open water, and woody wetland cover types, respectively (Fig 1, Table 4).

**Table 4:** Parameter estimates and 95% confidence intervals (CI) for the top-ranking model of eastern diamondback rattlesnake habitat selection at the home range scale in the South Carolina Coastal Plain tidewater region.

Parameter^a^	Estimate	SE	Lower 95% CI	Upper 95% CI	*P* > |t|
Intercept	-6.230	1.103	-8.837	-3.622	<0.001
Cover (FOR)	-1.664	0.227	-2.110	-1.219	<0.001
Cover (FP)	-1.717	0.488	-2.674	-0.761	<0.001
Cover (HW)	-1.247	0.271	-1.778	-0.717	<0.001
Cover (OW)	-1.935	0.655	-3.220	-0.650	0.003
Cover (WW)	-1.308	0.210	-1.718	-0.897	<0.001
Aspect	1.568	0.243	1.092	2.044	<0.001
Elev	-0.037	0.032	-0.101	0.026	0.247

Pine savanna (PS) was the reference for cover. FOR = forested, FP = wildlife food plot, HW = herbaceous wetland, OW = open water, WW = woody wetland.

^a^ Intercept df = 7; variable df = 1414

### Within Home Range Selection

**Fig 2:**
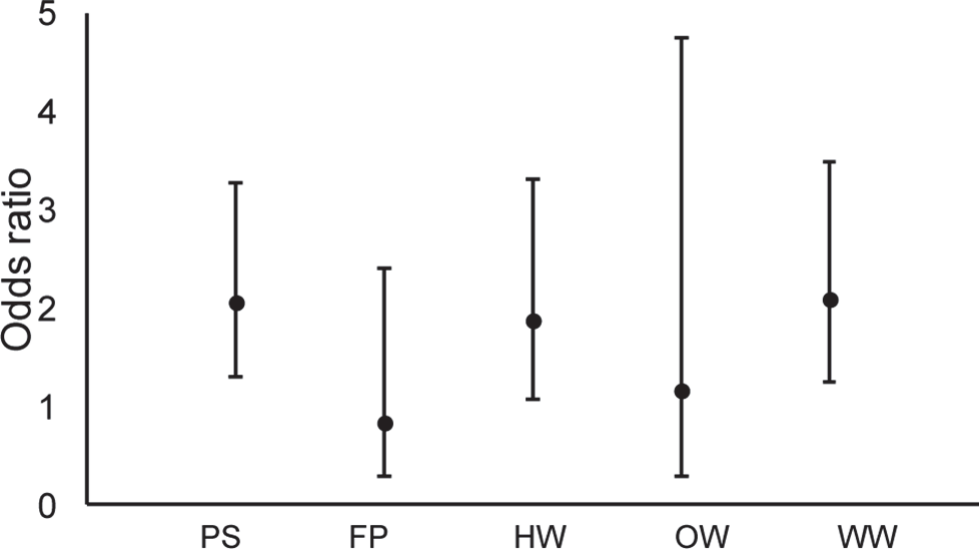
Odds ratios by cover type for eastern diamondback rattlesnake habitat selection at the within-home range scale in the South Carolina Coastal Plain tidewater region. Reference = forest; PS = pine savanna, FP = wildlife food plot, HW = herbaceous wetland, OW = open water, WW = woody wetland.

At the WHR scale, there were two supported models of EDB habitat selection (Table 3). Contrary to our predictions, both vegetation and topographic characteristics had significant influence on WHR selection. Cover type was a significant predictor, indicating a strong negative association with forested cover alone (Table 5). While our LiDAR-derived canopy cover was not a significant predictor, the LiDAR-derived measure of ground cover was significantly associated with EDB WHR selection. Not only was aspect a significant influence on habitat selection, with snakes selecting southwesterly-facing aspects (model-averaged β = 1.202, LCL = 0.740, UCL = 1.670), but it was also the most important predictor at this scale, contributing to almost 100% of model weights. Elevation, canopy cover, ground cover, and cover followed, contributing to 58%, 55%, 48%, and 45% of model weights, respectively. Sex, however, only accounted for 32% of model weights. Odds ratios indicated that the odds of rattlesnakes using forest were significantly less than the odds of rattlesnakes using other cover types within their home ranges (Fig 2, Table 5).

**Table 5:** Parameter estimates and 95% confidence intervals (CI) for top-ranking models of eastern diamondback rattlesnake habitat selection at the within-home range scale in the South Carolina Coastal Plain tidewater region.

Parameter	Estimate	SE	Lower 95% CI	Upper 95% CI	*P* > |t|
Model 1^a^					
Intercept	-4.527	1.163	-7.276	-1.778	0.006
FOR	-0.722	0.236	-1.186	-0.259	0.002
FP	-0.904	0.508	-1.900	0.093	0.075
HW	-0.094	0.281	-0.645	0.457	0.738
OW	-0.572	0.714	-1.973	0.829	0.423
WW	0.015	0.232	-0.441	0.471	0.948
Aspect	1.173	0.236	0.710	1.636	<0.001
Elevation	-0.013	0.035	-0.081	0.056	0.720
Model 2^b^					
Intercept	-4.505	0.741	-6.258	-2.753	<0.001
Cancov	-0.492	0.417	-1.311	0.327	0.239
Groundcov	4.364	1.899	0.637	8.091	0.022
Aspect	1.231	0.236	0.769	1.694	<0.001

Pine savanna (PS) was the reference for cover. FOR = forested, FP = wildlife food plot, HW = herbaceous wetland, OW = open water, WW = woody wetland.

^a^ Intercept df = 7, variable df = 902

^b^ Intercept df = 7, variable df = 906

## Discussion

Although we detected some differences in the strength of selection, our EDB habitat analyses were largely consistent across scales and further demonstrated the species’ association with open canopy habitats (i.e., savannas). However, at the HR scale EDBs exhibited a significant negative association with tidal marshes. Contrary to our expectations, EDBs do not appear to use tidal marshes as a surrogate habitat at the HR scale, and the significant negative association excludes using landscape patches defined by tidal marshes as suitable EDB habitat at this scale. The results of our HR analysis are an important consideration for large-scale conservation strategies because they preclude using tidal marsh as ‘suitable’ EDB habitat when modeling the species potential distribution or estimating available habitat.

At the HR scale, EDBs exhibited significant negative associations with all cover types other than pine savanna, likely reflecting selection of savanna ecosystems at the landscape scale. This result is consistent with other published studies of EDB habitat selection. Landscape-scale EDB occupancy rates on coastal barrier islands are negatively influenced by maritime forest area, and positively influenced by secondary dune habitat, a cover type similar in structure to pine savannas [51]. Likewise, inland EDB populations are positively associated with pine savannas and flatwoods [21, 27], and negatively associated with forested cover types [21] at large scales. These studies demonstrate the importance of savanna ecosystems at landscape scales, and may reflect the extent to which both EDBs and savannas historically occurred within southeastern USA. When coupled with species’ longevity [52] it seems evident that EDB spatial distributions may serve as a signal of high-integrity habitat at broad scales.

We failed to detect a positive association with any cover type when modeling WHR selection. Instead, EDBs distributions at our study site appeared to be driven by a negative association with forested cover within the home range, as are inland populations [21, 27]. Stohlgren (2013) also failed to detect any influence of barrier island landscape composition on EDB occupancy at smaller scales. Our failure to document other significant habitat relationships at this scale allows for the possibility that EDBs are using surrogate habitats of similar structure (e.g., marsh and food plots), within their home ranges. Therefore, although our HR analysis did not support tidal marsh as a surrogate habitat, marsh may still provide resources for EDBs at smaller scales.

Our LiDAR-derived estimates of canopy closure did not provide valuable habitat metrics at either scale of analysis. The study site was predominately open-canopied and a lack of heterogeneity likely reduced the utility of our LiDAR-derived habitat metric. Other model parameters support the importance of canopy cover for delineating EDB habitat, thus an integrated measure of ecological structure and function, such as cover type, may be more appropriate when defining habitat patches and characterizing the EDB’s landscape. For example, an integrated approach could account for more than simply the open-canopy structure of a range of cover types (dry sandhills, coastal dunes, mesic flatwoods) within the EDB’s geographic range. For example, the relative availability of specific prey items could also be modeled.

LiDAR-derived ground cover was positively associated with EDB habitat selection at the WHR scale. This result is consistent with microhabitat selection by inland EDBs [22], and may reflect selection of shrubby microhabitats for foraging within their home ranges. As open canopy ecosystems with a diverse groundcover, including shrub patches [53], pine savannas support a number of ground-dwelling prey [54]. For example, large prey species such as cotton rats are associated with shrub cover and low canopy cover [55] and with high grass density [56]. However, we found a non-significant negative association of EDBs with fields within their home ranges, similarly to other studies [27, 38]. Predation risk from insufficient cover might account for rattlesnake avoidance of fields, or snakes may be simply using the edges around fields. Waldron et al. (2006a) found a significantly positive association of EDBs with fields at the HR scale. These fields were planted with corn and likely attracted greater numbers of prey, whereas many of those in the present study were fallow. Fields may therefore be an important component of EDB home ranges when crops that attract prey function as predator subsidization.

We predicted that topography would provide insight into EDB distributions only at large scales, and thus inform our understanding of home range selection. Interestingly, however, aspect was a significant predictor at both scales. Other rattlesnake species are known to use hibernacula in south- or southwest-facing locations [57–59], yet there is no information on the role of aspect in EDB habitat selection. It is possible that our results reflect EDB thermoregulatory needs [60]; however, this is unlikely because the majority of our radio telemetry observations were recorded during the active season. Ambient conditions during the active season in this region considerably reduce the need for basking behavior, which we suspect is more important during winter when animals spend intermittent periods on the surface during the dormant season. Elevation was a statistically important but non-significant predictor at both scales. This is likely due to the low variation in elevation at our study site (~12 m), necessitating further study at sites with more prominent topography to elucidate the role of elevation in EDB habitat selection.

Loss of habitat, particularly of longleaf pine savannas, is a dominant factor in EDB decline [21–23]. Our study adds to previous research that underscores the importance of pine savannas for maintaining EDB populations at large scales. Eastern diamondback rattlesnake conservation is therefore fundamentally linked with management and restoration of remnant savanna landscapes on a regional level [22]. Fire exclusion or changed fire regimes in pine savannas support invasion of more mesic plant species that modify the fuel structure, alter fire frequency, and destabilize the pine savanna state [61–63]. These disturbances have consequences for EDB prey abundance and distribution [64–66] and thermoregulatory opportunities [67]. Thus, despite potential for additional habitats to serve as surrogates at smaller scales, pine savanna significance at both scales underscores the importance of pine savanna integrity to EDB viability.

Our classification approach did not support tidal marsh or agricultural fields as valid surrogate habitats for tidal-region EDB populations at the landscape scale. However, within their home ranges, EDBs at our study site appear to use structurally similar open-canopy habitats that we suspect may be satisfying their trophic niche requirements by supplying large-bodied prey. In coastal areas, these patches include marsh and brackish impoundments that support large ground-dwelling mammals. These results demonstrate the utility of multi-scale examinations of surrogate habitat potential that may inform approaches to animal conservation and management.

## Supporting Information

S1 Table Vegetative and topographic characteristics of used and random locations in analyses of EDB habitat selection. (XLSX)

## References

[1] Chase JM, Leibold MA (2003) *Ecological niches*: *linking classical and contemporary approaches* Chicago: University of Chicago Press. 221 pp.

[2] Dunning JB, Danielson BJ, Pulliam HR (1992) Ecological processes that affect populations in complex landscapes. Oikos 65: 169–175.

[3] Merola-Zwartjes M, DeLong JP (2005) Avian species assemblages on New Mexico golf courses: surrogate riparian habitat for birds? Wildl. Soc. B. 33: 435–447.

[4] Berndt LA, Brockerhoff EG, Jactel H. (2008) Relevance of exotic pine plantations as a surrogate habitat for ground beetles (Carabidae) where native forest is rare. Biodivers. Conserv. 17: 1171–1185.

[5] Rysava-Novakova M, Ondrackova M, Jurajda P (2009) The importance of surrogate habitats in lowland river floodplains for fish community composition. Fisheries Manag. Ecol. 16: 468–477.

[6] Burt WH (1943) Territoriality and home range concepts as applied to mammals. J Mammal 24: 346–352.

[7] Hall LS, Krausman PR, Morrison ML (1997) The habitat concept and a plea for standard terminology. Wildl Soc B 25: 173–182.

[8] Powell RA, Mitchell MS (2012) What is a home range? J Mammal 93: 948–958.

[9] Morris DW (1987) Ecological scale and habitat use. Ecol 68: 362–369.

[10] Wiens JA, Rotenberry JT, van Horne B (1987) Habitat occupancy patterns of North American shrubsteppe birds: the effects of spatial scale. Oikos 48: 132–147.

[11] Wiens JA (1989) Spatial scaling in ecology. Funct Ecol 3: 385–397.

[12] Boyce MS (2006) Scale for resource selection functions. Diverse Distrib 12: 269–276.

[13] Johnson DH (1980) The comparison of usage and availability measurements for evaluating resource preference. Ecol 61: 65–71.

[14] Jose S, Jokela EJ, Miller DL (2006) The longleaf pine ecosystem: ecology, silviculture, and restoration New York: Springer-Verlag. 438 pp.

[15] Ware S, Frost C, Doerr PD (1993) Southern mixed hardwood forest: the former longleaf pine forest In: Martin WH, Boyce SG, Echternacht AC, editors. Biodiversity of the southeastern United States: lowland terrestrial communities. New York: John Wiley and Sons pp. 447–493.

[16] Frost CC (1993) Four centuries of changing landscape patterns in the longleaf pine ecosystem. In: Hermann SM, editors. Proceedings of the Tall Timbers Fire Ecology Conference. Tallahassee: Tall Timbers Research Station. pp. 17–43.

[17] Frost CC (2006) History and future of the longleaf pine ecosystem In: Jose S, Jokela EJ, Miller DL, editors. The longleaf pine ecosystem: ecology, silviculture, and restoration. New York: Springer-Verlag pp. 9–42.

[18] Means DB (2006) Vertebrate faunal diversity of longleaf pine ecosystems In: Jose S, Jokela EJ, Miller DL, editors. The longleaf pine ecosystem: ecology, silviculture, and restoration. New York: Springer-Verlag pp. 157–213.

[19] Sorrie BA, Weakley AS (2006) Conservation of the endangered *Pinus palustris* ecosystem based on Coastal Plain centres of plant endemism. Appl Veg Sci 9: 59–66.

[20] Timmerman WW, Martin WH (2003) Conservation guide to the eastern diamondback rattlesnake, *Crotalus adamanteus*. Soc Study Amph Reptiles Herpetol Circ No 32.

[21] Waldron JL, Bennett SH, Welch SM, Dorcas ME, Landhan JD, Kalinowsky W (2006a) Habitat specificity and home range size as attributes of species vulnerability to extinction: a case study using sympatric rattlesnakes. Anim Conserv 9: 414–420.

[22] Waldron JL, Welch SM, Bennett SH (2008) Vegetation structure and the habitat specificity of a declining North American reptile: A remnant of former landscapes. Biol Conserv 141: 2477–2482.

[23] Martin WH, Means DB (2000) Distribution and habitat relationships of the eastern diamondback rattlesnake (*Crotalus adamanteus*). Herpetological Nat Hist 7: 9–34.

[24] United States Fish and Wildlife Service. 2012 Endangered and threatened wildlife and plants: 90-day finding on a petition to list the eastern diamondback rattlesnake as threatened. Federal Register 77: 27403–27411.

[25] Gibbons JW, Scott DE, Ryan TJ, Buhlmann KA, Tuberville TD, Metts BS, et al (2000) The global decline of reptiles, de´ ja`vu amphibians. BioScience 50: 653–666.

[26] Waldron JL, Lanham JD, Bennett SH (2006b) Using behaviorally-based seasons to investigate canebrake rattlesnake (*Crotalus horridus*) movement patterns and habitat selection. Herpetologica 62: 389–398.

[27] Hoss SK, Guyer C, Smith LL, Schuett GW (2010) Multiscale influences of landscape composition and configuration on the spatial ecology of eastern diamond-backed rattlesnakes (*Crotalus adamanteus*). J Herpetol 44: 110–123.

[28] Waldron JL, Welch SM, Holloway J, Mousseau TA (2013b) Using occupancy models to examine human-wildlife interactions. Hum Dimens Wildl 18: 138–151.

[29] ESRI (2011) Redlands: www.esri.com/software/arcgis.

[30] Seaman DE, Powell RA (1996) An evaluation of the accuracy of kernel density estimators for home range analysis. Ecol 77: 2075–2085.

[31] Jones MC, Marron JS, Sheather SJ (1996) A brief survey of bandwidth selection for density estimation. J Am Statist Assoc 91: 401–407.

[32] Gitzen RA, Millspaugh JJ, Kernohan BJ (2006) Bandwidth selection for fixed-kernel analysis of animal utilization distributions. J of Wildl Manage 70: 1334–1344.

[33] Solla DE, Shane R, Bonduriansky R, Brooks RJ (1999) Eliminating autocorrelation reduces biological relevance of home range estimates. J Anim Ecol 68: 221–234.

[34] Swihart RK, Slade NA (1997) On testing for independence of animal movements. J Agr Biol Envir St 2: 48–63.

[35] Borger L, Franconi N, de Michele G, Gantz A, Meschi F, Manica A, et al (2006) Effects of sampling regime on the mean and variance of home range size estimates. J Anim Ecol 75: 1393–1405. 17032372 10.1111/j.1365-2656.2006.01164.x

[36] Kochanny CO, Delgiudice GD, Fieberg J (2009) Comparing global positioning system and very high frequency telemetry home ranges of white-tailed deer. J Wildl Manage 73: 779–787.

[37] Row JR, Blouin-Demers G (2006) Kernels are not accurate estimators of home-range size for herpetofauna. Copeia 4: 797–802.

[38] Timmerman WW (1995) Home range, habitat use, and behavior of the eastern diamondback rattlesnake (*Crotalus adamanteus*) on the Ordway Preserve. Bull Fla Mus Nat Hist 38: 127–158.

[39] Manly BF, McDonald L, Thomas D, McDonald TL, Erickson WP (2002) Resource selection by animals: statistical design and analysis for field studies Dordrecht: Kluwer Academic Publishers. 221 pp.

[40] Magellan (2005) Magellan Explorist GPS <http://support.magellangps.com/support/assets/manuals/eXplorist_500_en.pdf>. 112 pp.

[41] Zimble DA, Evans DL, Carlson GC, Parker RC, Grado SC, Gerard PD (2003) Characterizing vertical forest structure using small-footprint airborne LiDAR. Remote Sens Environ 87: 171–182.

[42] Listopad CMCS (2011) Applications of airborne and portable lidar in the structural determination, management, and conservation of southeastern U.S. pine forests. PhD dissertation, University of Central Florida.

[43] Lim K, Treitz P, Wulder M, St-Onge B, Flood M (2003) LiDAR remote sensing of forest structure. Prog Phys Geog 27: 88–106.

[44] Fry J, Xian G, Jin S, Dewitz J, Homer C, Yang L, et al (2011) Completion of the 2006 National Land Cover Database for the Conterminous United States. Photogramm. Eng. and Remote Sens. 77: 858–864.

[45] Jenness Enterprises (2013) DEM Surface Tools for ArcGIS.

[46] Ford WM, Chapman BR, Menzel MA, Odom RH (2002) Stand age and habitat influences on salamanders in Appalachian cove hardwood forests. For Ecol Manage 155: 131–141.

[47] Dillard LO, Russell KR, Ford WM (2008) Site-level habitat models for the endemic, threatened Cheat Mountain salamander (*Plethodon nettingi*): The importance of geophysical and biotic attributes for predicting occurrence. Biodivers Conserv 17: 1475–1492.

[48] SAS Institute (2011) SAS v. 9.3. Cary: SAS Institute.

[49] Burnham KP, Anderson DR (2002) Model selection and multimodel inference: a practical information-theoretic approach New York: Springer-Verlag. 488 pp.

[50] Raudenbush SW, Yang ML, Yosef M (2000) Maximum likelihood for generalized linear models with nested random effects via high-order, multivariate Laplace approximation. J Comput Graph Stat 9: 141–157.

[51] Stohlgren KM (2013) Eastern diamondback rattlesnakes: using occupancy and population models to direct management. M.S. thesis, University of Georgia.

[52] Waldron JL, Welch SM, Bennett SH (2013a) Life history constraints contribute to the vulnerability of a declining North American rattlesnake. Biol Conserv 159: 530–538.

[53] Platt WJ (1999) Southeastern pine savannas In: Anderson RC, Fralish JS, Basking JM, editors. Savannas, barrens, and rock outcrop plant communities of North America. Cambridge: Cambridge University Press pp. 23–51.

[54] Golley FB, Gentry JB, Caldwell LD, Davenport LB Jr. (1965) Number and variety of small mammals on the AEC Savannah River plant. J Mammal 46: 1–18.

[55] Bowne DR, Peles JD, Barrett GW (1999) Effects of landscape spatial structure on movement patterns of the hispid cotton rat (*Sigmodon hispidus*). Landscape Ecol 14: 53–65.

[56] Kincaid WB, Cameron GN (1985) Interactions of cotton rats with a patchy environment: dietary responses and habitat selection. Ecol 66: 1769–1783.

[57] Bertram N, Larsen KW, Surgenor J (2001) Identification of critical habitats and conservation issues for the Western Rattlesnake and Great Basin Gopher Snake within the Thompson-Nicola region of British Columbia British Columbia Ministry of Water Land and Air Protection and the Habitat Conservation Trust Fund British Columbia, Canada. 53 pp.

[58] Clark RW, Brown WS, Stechert R, Zamudio KR (2008) Integrating individual behaviour and landscape genetics: the population structure of timber rattlesnake hibernacula. Mol Ecol 17: 719–730. 18028304 10.1111/j.1365-294X.2007.03594.x

[59] Gienger CM, Beck DD (2011) Northern Pacific Rattlesnakes (*Crotalus oreganus*) use thermal and structural cues to choose overwintering hibernacula. Can J Zool 89: 1084–1090.

[60] Huey RB (1991) Physiological consequences of habitat selection. Am Nat 13: S91–S115.

[61] Glitzenstein JS, Platt WJ, Streng DR (1995) Effects of fire regime and habitat on tree dynamics in north Florida longleaf pine savannas. Ecol Monogr 65: 441–476.

[62] Beckage BW, Platt WJ, Gross LJ (2009) Vegetation, fire, and feedbacks: a disturbance-mediated model of savannas. Am Nat 174: 805–818 doi: 10.1086/648458 19860540 10.1086/648458

[63] Kreye JK, Varner JM, Hiers JK, Mola J (2013) Toward a mechanism for eastern North American forest mesophication: differential litter drying across 17 species. Ecol Appl 23: 1976–1986. 24555322 10.1890/13-0503.1

[64] Schweiger EW, Diffendorfer JE, Holt RD, Pierotti R, Gaines MS (2000) The interaction of habitat fragmentation, plant, and small mammal succession in an old field. Ecol Monogr 70: 383–400.

[65] Torre I, Diaz M (2004) Small mammal abundance in Mediterranean post-fire habitats: a role for predators? Acta Oecologica 25: 137–142.

[66] Jenkins CL, Peterson CR (2008) A trophic-based approach to the conservation biology of rattlesnakes: Linking landscape disturbance to rattlesnake populations In Hayes WK, Beaman KR, Cardwell MD, Bush SP, editors. The biology of rattlesnakes. Loma Linda: Loma Linda University Press pp. 265–274.

[67] Pringle RM, Webb JK, Shine R (2003) Canopy structure, microclimate, and habitat selection by a nocturnal snake, *Hoplocephalus bungaroides* . Ecol 84: 2668–2679.

